# Quality Assessment of Pork and Turkey Hams Using FT-IR Spectroscopy, Colorimetric, and Image Analysis

**DOI:** 10.3390/foods7090152

**Published:** 2018-09-15

**Authors:** Vassilia J. Sinanoglou, Dionisis Cavouras, Dimitrios Xenogiannopoulos, Charalampos Proestos, Panagiotis Zoumpoulakis

**Affiliations:** 1 Laboratory of Chemistry, Analysis and Design of Food Processes, Department of Food Science and Technology, University of West Attica, Ag. Spyridonos 12243, Egaleo, Greece; xenodimi@hotmail.com; 2 Medical Image and Signal Processing Laboratory, Department of Biomedical Engineering, University of West Attica, Ag. Spyridonos 12243, Egaleo, Greece; cavouras@teiath.gr; 3 Laboratory of Food Chemistry, Department of Chemistry, National and Kapodistrian University of Athens, 15784 Athens, Greece; harpro@chem.uoa.gr; 4 Institute of Biology, Medicinal Chemistry and Biotechnology, National Hellenic Research Foundation, 48, Vas. Constantinou Ave., 11635 Athens, Greece

**Keywords:** pork and turkey ham, color, image analysis, Fourier Transform Infrared Spectroscopy, shelf life

## Abstract

The implementation of fast and nondestructive methods in meat products and colds cuts have become increasingly important to evaluate their quality in relation to different factors such as origin, type of processing, freshness, adulteration, and authenticity. In this study, Fourier Transform Infrared Spectroscopy (FT-IR), colorimetric, and image-analysis methods were implemented to characterize and classify ham cold cuts in terms of meat type, processing, and shelf life during refrigerated storage. Two types of commercial hams (made from pork and turkey) and three types of processing (boiled, smoked, and roasted) were selected. By using the most appropriate color parameters, a*, h, and C*, as well as the textural features’ angular second moment, long-running emphasis, and standard deviation of image intensity from the hams’ images, high-classification values for the different ham samples were achieved. The FT-IR analysis revealed the presence of absorbance bands of proteins, triglycerides, fatty acids, and carbohydrates with different intensities according to meat type and processing. Refrigeration storage caused significant alterations of color parameters and a partial degradation of triglycerides and proteins. Moreover, the image-analysis findings indicated that storage period caused significant degradation of ham images relating to local linearity, and structural and textural continuum.

## 1. Introduction

Ham is produced from the meat of the back leg of pork and preserved through salting, boiling, smoking, and dry and wet curing. However, the term ham may legitimately be used for other varieties of meat, such as turkey and chicken, provided the meat is taken from the bird’s chest or leg [1]. There is a wide range of ham products made from countless meat products, and different processing methods [2]. Hams are available fresh, salted, and sometimes smoked. There are many different varieties of hams, which vary according to the methods used in the curing process. The curing process is used to maintain them, develop a darker color, and enhance their taste. There are three main categories of ham: fresh, dry-cured, and wet or brine-cured ham [3,4].

The great interest of consumers towards the safety and quality of cold cuts makes it imperative to develop and apply rapid, low-cost, and nondestructive methods. Several techniques have been applied for the characterization and discrimination of cold cuts. Digital-image analysis has been previously utilized for the evaluation of the quality of cold meats [5], the classification of presliced pork- and turkey-ham qualities [1], the assessment of changes of the histological parameters of pork muscles [6], and the quality assessment of fresh meats [7]. Moreover, color measurement has been used for many applications towards cold cuts’ quality assessment [8]. Furthermore, Fourier transform infrared (FT-IR) spectroscopy is employed to register the absorbance bands of different compounds in meat products [9,10,11,12].

Given the fact that digital image analysis, color measurement, and Fourier Transform Infrared Spectroscopy (FT-IR) have had increasing interest related to quality control, authenticity, and safety of food items, their combination could be a powerful tool to evaluate the quality of hams. Therefore, the aim of the present research was to characterize hams according to meat type (pork and turkey), processing (boiled, smoked, roasted), and shelf-life during refrigerated storage. For the implementation of the above experimental objectives, FT-IR, color, and image-analysis methods were applied. The above nondestructive methods could be applied to a variety of food products to monitor their quality and chemical stability during storage.

## 2. Materials and Methods

### 2.1. Ham Samples

Five types of commercial hams (30 slices per type) made from pork (boiled, smoked, and roasted) and turkey (boiled and smoked) were purchased from different producers. The slices were packed in moisture-impermeable polyethylene containers, sealed, and stored at 4 °C. The samples were analyzed every 3 days for a total of 3 weeks.

### 2.2. Color Measurement

Color values L* (lightness), a* (redness/greenness), b* (yellowness/blueness), C* (chroma), h* (hue angle in degrees), and DE* (total color measurement) were measured with a tristimulus chromatometer (model CR-400, Minolta, Tokyo, Japan) calibrated with a white standard plate (L*: 97.83, a*: −0.45, b*: +1.88). Five random readings per sample were taken and averaged.

### 2.3. Image Acquisition

**Figure 1:**
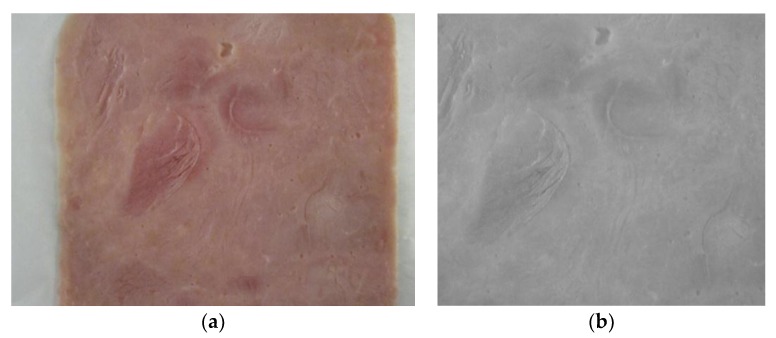
(**a**) Digital image of boiled-pork slice, and (**b**) grayscale sample used in textural-feature calculation.

Ham cold cuts were digitally photographed using the Sony DSCW800/B digital camera (Sony Europe Limited, Edinburgh, United Kingdom), placed 25 cm from the ham slice (Figure 1a). The imaging setup was housed within a cover to isolate external light sources, and appropriate lighting conditions were selected to safeguard constant illumination conditions. Images of ham slices were acquired at lens aperture f = 4.6 and 1280 × 720 pixels resolution. The images were saved in jpeg format. From each image, 22 textural features were calculated. Those features quantify textural properties of each ham slice’s surface. Textural features were computed from the grayscale version of the images (Figure 1b).

The goal was to assess differences in ham-slice texture between types of ham (pork and turkey) and between types of processing (boiled and smoked). The grayscale version of images was used for estimating textural changes occurring with storage time. Four features were calculated from the grayscale-image histogram (first-order statistics: mean value, standard deviation, skewness, and kurtosis). Thirteen features were computed from the image’s co-occurrence matrix [13] (second-order statistics: angular second moment, contrast, correlation, sum of squares, inverse difference moment, entropy, sum entropy, sum average, sum variance, difference variance, difference entropy, information measure correlation I, information measure correlation II). Five features were computed from the image’s run-length matrix [13] (second-order statistics: short-run emphasis, long-run emphasis, gray-level nonuniformity, run-length nonuniformity, run percentage) [13]. In addition, the 6 color parameters (L*, a*, b*, c*, h*, DE*) were employed as features. Each image of a ham slice was thus represented by 28 feature values. These values were held into a 28-long vector. Different classes were formed, each containing the feature vectors of specific types of ham and of a different processing method. These classes were used in the discriminant and regression analyses. To avoid bias, all features were normalized to zero mean and unit standard deviation for use in the discriminant analysis, employing the following function: f¯i=(fi−μ)σ, where μ and σ are the mean and standard deviations of the feature $f_{i}$, both calculated over all patterns of the classes involved in the discrimination process (e.g., turkey boiled vs. turkey smoked), and ${\bar{f}}_{i}$ is the normalized version of feature $f_{i}$.

### 2.4. Discriminant Analysis

The class-discrimination problem was viewed as a pattern-recognition problem, in that discrimination is mainly performed by a classifier. The aim was to choose among the large number of readily available classifiers the one classifier that would be fast in execution, robust in performance, and would achieve high discrimination accuracy. Several classifiers were tested (Artificial Neural Network, Bayesian, Linear Discriminant Analysis, Logistic Regression, Nearest Neighbor, Probabilistic Neural Network, and Support Vector Machines). The precision of those classifiers was tested on the available data of the present study. Most of those classifiers are available as functions in Matlab. To test the classifiers, first, all possible combinations of features were formed (i.e., combinations of 1, 2, 3, … etc. features). The classifier was designed by using a feature combination and the classifier’s precision was tested for discriminating between the classes by means of the leave-one-out (LOO) evaluation method. According to LOO, the classifier is designed by all class vectors but 1, which is left out. The latter is used as input to the classifier to be assigned into 1 of 2 or 3 classes. The left-out vector is reinserted into its class, the next one is left out, the classifier is redesigned, and the process is repeated until all feature vectors are processed by the classifier and are assigned to a particular class. Since the true class of each feature vector is a priori known, a truth table is built indicating the correctly and incorrectly classified feature vectors. In this way, the best design, which achieved the highest discrimination accuracy with the least number of features, was determined. Each classifier was subjected to that evaluation process and it was found that the Probabilistic Neural Network (PNN) [14] classifier with Gaussian kernel proved efficient to design and accurate enough to adopt for discriminating the dataset of the present study.

### 2.5. Nonlinear Regression Analysis

Nonlinear regression analysis was employed using a second-order polynomial to fit the variation of features with storage time. At preset 3-day time intervals, each ham slice was photographed and its features vector was computed. For each class (e.g., boiled turkey), nonlinear regression analysis was used to describe in one figure the variation of each feature with storage time. Sprearman’s correlation coefficient was used to evaluate the goodness of fit of the polynomial (*r* > 0.2) to the data. Matlab functions (corr and polyfit) were employed for the nonlinear regression analysis.

### 2.6. FT-IR Spectra Acquisition

FT-IR spectra were obtained using a Nicolet Nexus FT-IR spectrophotometer (Thermo Electron Corporation, Waltham, MA, USA) with a resolution of 4 cm^−1^ at 32 scans. A small quantity of homogenized ham samples (0.001 g) was directly deposited between 2 well-polished KBr disks, creating a thin film. Triplicated spectra were recorded for all the samples. Spectra were scanned in the absorbance mode from 4000 to 500 cm^−1^, at room temperature (24 °C), and the data were handled with EZ OMNIC 7.3 software (Thermo Electron Corporation, Waltham, MA, USA).

### 2.7. Statistical Analysis

All statistical calculations regarding color measurement and FT-IR spectra interpretation were performed using SPSS statistical analysis software for Windows (IBM SPSS Statistics, version 19.0, Chicago, IL, USA).

## 3. Results and Discussion

### 3.1. Classification of Hams According to Meat Type and Processing, Using Color-Measurement, FT-IR Spectra-Interpretation, and Textural-Image Analysis Methods

Color is a significant indicator of meat quality, because it is one of the most important features influencing the evaluation of meat by the consumer [2,15]. In the food industry, the most popular numerical color space system is the CIE L*, a* and b*.

Results of color measurement are shown in Table 1. Regarding turkey hams, the smoking process led to a reduction (*p* < 0.05) of lightness (L*) and of total color change (DE*) values and an increase of hue (h) value. The smoking treatment, however, did not affect yellowness (b*) (*p* > 0.05).

Concerning pork hams, color measurements on the surface of boiled and smoked ones revealed no statistical differences (*p* > 0.05) between the average color values L*, C*, h, ΔE*, a* and b*. In contrast, roasted process led to an increase (*p* < 0.05) of chroma (relative saturation, C*) of redness (a*), of yellowness (b*) and of total color change (DE*) values.

Color values L*, h, a* and b* showed significant differences (*p* < 0.05) between pork- and turkey-ham samples of the same processing. Specifically, turkey-ham slices revealed almost double b* values and nearly half a* values compared to pork hams. Dvorak et al. [16] reported that redness is the most important color parameter in assessing the quality of pork ham. Moreover, Garcia-Esteban et al. [17] reported that a* is the most sensitive parameter, featuring the red color and color stability of meat products. Furthermore, turkey-ham slices were characterized by significantly higher (*p* < 0.05) lightness (L*) and hue (h) values compared to pork ones, with turkey fillets having almost twice the hue value (h) of the pork.

Pattern-recognition methods were employed using textural features from the hams’ images, in order to highlight an initial grouping of the samples, depending on meat type (pork and turkey) and processing (boiled, smoked, or roasted), and to gain suitable information for the selection of the most appropriate features for the potential separation among groups.

**Figure 2:**
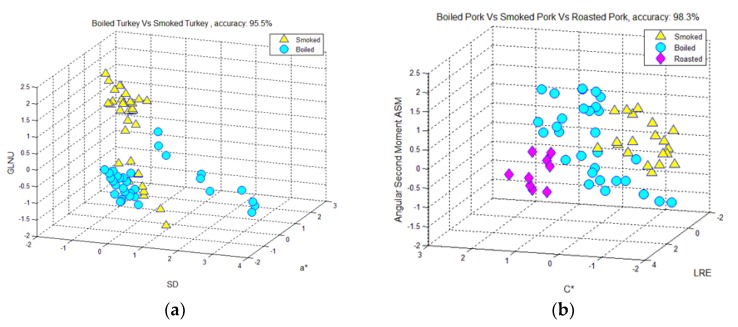
Scatter diagram displaying the discrimination, employing color parameters and textural features, between: (**a**) boiled- and smoked-turkey hams; and (**b**) boiled-, smoked-, and roasted-pork hams.

A scatter diagram (Figure 2a) showed that boiled turkey hams were separated from smoked ones with 95.5% overall discrimination accuracy, via the features of standard deviation (SD), a* and gray-level nonuniformity (GLNU). SD is computed from the image histogram and it quantifies textural inhomogeneity. Feature a* is a color feature and it quantifies image redness/greenness. GLNU is a second-order statistic [13,18] and it quantifies unevenness in the distribution of image structures throughout the grey levels. This three-feature combination was used in the design of a high-performance pattern-recognition system that correctly classified 32 out of 34 boiled turkey slices (two slices were wrongly assigned to the smoked-turkey class), and 31 out of 32 smoked turkey slices (one slice was wrongly classified to the boiled-turkey class). As shown in Figure 2a, smoked-turkey slices, in comparison with boiled-turkey slices, had higher GLNU but lower a* and SD values.

As shown in the scatter diagram of Figure 2b, the three types of pork hams were separated with 98% overall precision. This was realized employing three features, one feature from the color attributes, chroma (C*), and two textural features, angular second moment (ASM) from the co-occurrence matrix, and long-run emphasis (LRE) from the run-length matrix. This three-feature combination provided a high-precision pattern-recognition system that correctly classified all but one boiled-pork slice. ASM quantifies image homogeneity, and LRE indicates the presence of large structures in the image. ASM was higher in boiled-pork slices, LRE was higher in smoked-pork-ham slices, and chroma C* was higher in roasted-pork slices.

**Figure 3:**
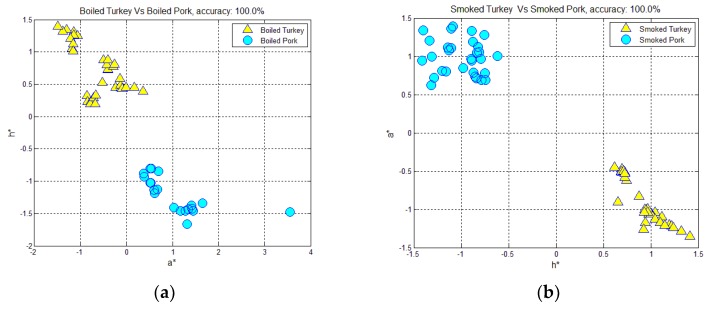
Scatter diagram displaying the discrimination, employing the color parameters and textural features, between: (**a**) boiled turkey and pork hams; (**b**) smoked turkey and pork hams.

Concerning the meat type, the scatter diagrams of Figure 3a,b showed the adequate separation between boiled turkey and pork hams, as well as between smoked turkey and pork hams via hue (h) and redness (a*) values, with 100% overall precision. Therefore, these results suggest that the meat type altered the color parameters, allowing for clear distinction.

**Figure 4:**
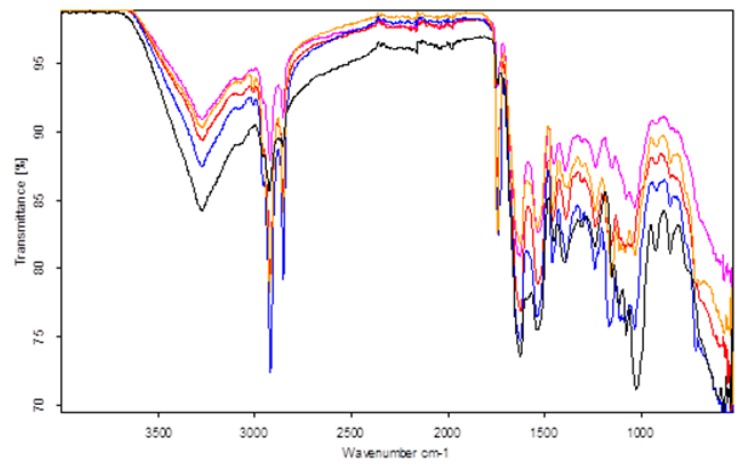
FFourier Transform Infrared Spectroscopy (FT-IR) spectra of boiled-turkey (pink), smoked-turkey (yellow), boiled-pork (red), roasted-pork (blue), and smoked-pork (black) hams.

Ham slices were further analyzed using FT-IR spectroscopy in the midinfrared region (4000–500 cm^−1^). All spectra (Figure 4) showed characteristic absorption bands associated with proteins, triglycerides, fatty acids, and carbohydrates. Characteristic bands of the spectra have been evaluated based on the literature data [9,10,11,12,19,20].

Specifically, the wide absorption band at 3500–3200 cm^−1^ is due to O-H and N-H symmetric stretching vibration of alcohols and amide/proteins, respectively. The band from 3100 to 3020 cm^−1^ is related to the vinyl C-H stretching vibrations of lipids. The strong absorption bands at 2950–2920 and 2852 cm^−1^ corresponds to methyl and methylene groups’ asymmetric and symmetric stretching vibrations of lipids, respectively. The characteristic band at 1744 cm^−1^ is assigned to C=O stretching of carbonyl group of triglycerides’ ester bond. Moreover, the strong absorption band at 1627 cm^−1^ is ascribed to C=O stretching vibration of amides, whereas the band at 1541 cm^−1^ to the combined vibration of the amino group (N-H) bending with the C-N stretching of proteins. According to Yu [21], the bands in the 1600–1700 and 1500–1560 cm^−1^ regions represent the protein primary features and are characterized as amide I and amide II, respectively. The weak absorbance band at 1530 cm^−1^ is related to the COO– asymmetric stretching vibration of fatty acids and proteins. The band at 1467 cm^−1^ is due to C-H bending of alkyl chain methylene of lipid moieties. The weak bands at 1450 and 1395 cm^−1^ correspond to C-H asymmetric and symmetric stretching vibrations, respectively, of protein moieties. The strong band from 1170 to 1154 cm^−1^ is attributed to C-O stretching of proteins and triglycerides. Phospholipids and nucleic acids are characterized by P=O asymmetric and symmetric stretching vibrations at 1240 and 1083 cm^−1^, respectively. The medium band at 1117 cm^−1^ is ascribed to C-H bending and deformation of fatty acids. The bands from 1040 to 1020 cm^−1^ are assigned to carbohydrate moieties and, in the case of meat products, it might indicate the presence of glycogen. The medium bands at 966 and 870 cm^−1^ are attributed to the out-of-plane deformation of CH=CH *trans* double bond and para-disubstituted aromatic derivatives, respectively.

From the interpretation of the FT-IR spectra of the ham samples (Table 2) (boiled turkey, smoked turkey, boiled pork, smoked pork, and roasted pork), the most interesting findings concerning the type of meat and processing are summarized below.

The spectra of pork samples, irrespective of processing type, showed absorbance bands of higher (*p* < 0.05) intensities at 2950–2920, 2852, and 1744 cm^−1^ than the spectra of the turkey samples. Therefore, it seems that pork hams have a higher (*p* < 0.05) lipid content compared to turkey hams. Moreover, since the band at 3100–3020 cm^−1^ was associated with the vibration of the olefin group, it could be estimated that turkey hams were characterized by higher content of unsaturated fatty acids than the pork hams. An important indication confirming the above finding was the presence of the band at 717 cm^−1^ only in the turkey-ham samples, which is associated with the out-of-plane bending vibration of the –CH=CH– group in unsaturated fatty acids [22]. Furthermore, pork hams have higher (*p* < 0.05) protein content compared to turkey hams, as the spectra of pork hams exhibited higher (*p* < 0.05) intensities at 1450, 1395, and 1170–1154 cm^−1^ than the ones of the turkey samples. In this sense, the adsorption bands at 1627 and 1541 cm^−1^, which are characteristics of the protein presence, showed a higher (*p* < 0.05) signal in the spectra of pork hams than in those of the turkey ones. Furthermore, according to Yu [21], if the amide I adsorption band, which characterizes the protein secondary structure, is located in the region, 1648–1658 and 1620–1640 cm^−1^ are associated with the α-helix and the β-sheet protein structure, respectively. Therefore, the major protein-structure content of pork- and turkey-ham samples is β-sheet. The spectra of the turkey hams presented significantly (*p* < 0.05) higher intensities at 1040–1020 cm^−1^ compared to the pork hams, which is indicative of higher carbohydrate content.

According to the findings of Wong et al. [23], when the ratio of C=O absorption at 1740 cm^−1^ to the P=O absorption at 1240 cm^−1^ is between 1.9 and 2.3, then the P=O bond is attributed to phospholipids, while outside this ratio to nucleic acids. In the spectra of all samples studied, the ratio was found to be approximately 1.3–1.4, which proves, on the basis of the above, that the P=O bond is attributed to nucleic acids.

High positive Pearson correlations were observed among lightness (L*), hue (h), and yellowness (b*), and the intensity values at 3100–3020 cm^−1^ (0.915, 0.847, and 0.712, respectively; *p* < 0.01) as well as the intensity values at 1040–1020 cm^−1^ (0.913, 0.971, and 0.769, respectively; *p* < 0.01). This result indicates a possible relationship among the above color parameters and the presence of olefin groups and high carbohydrate content, respectively. Moreover, significant positive correlations were determined between redness (a*) and the intensity values at 1627 and 1541 cm^−1^ (0.957 and 0.952, respectively; *p* < 0.01), which characterize the protein presence.

Concerning the type of processing, minor differences were identified from the interpretation of the FT-IR spectra of the ham samples. Therefore, only the spectra of the boiled-turkey hams showed significantly (*p* < 0.05) higher intensities than the smoked ones at the bands relating to lipid and protein content. Moreover, smoked hams, irrespective of meat type, presented significantly (*p* < 0.05) higher intensities at 966 cm^−1^ compared to the other hams, indicating the increase of *trans* double bonds.

### 3.2. Classification of Hams According to Shelf Life Using Color Measurement, FT-IR Spectra Interpretation, and Image-Texture Analysis Methods

**Figure 5:**
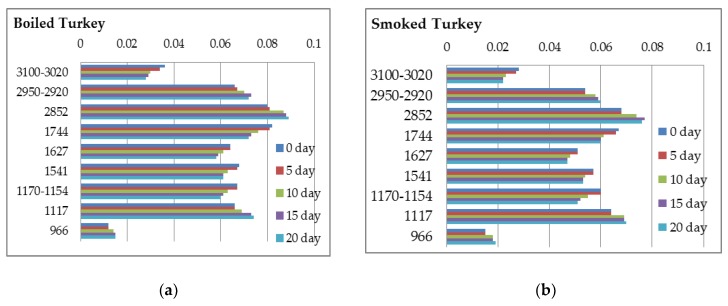
Spectral absorbance bands (intensities) (X-axis) at characteristic regions (cm^−1^) (Y-axis) during storage in refrigeration for twenty days: (**a**) boiled-turkey hams; (**b**) smoked-turkey hams.

**Figure 6:**
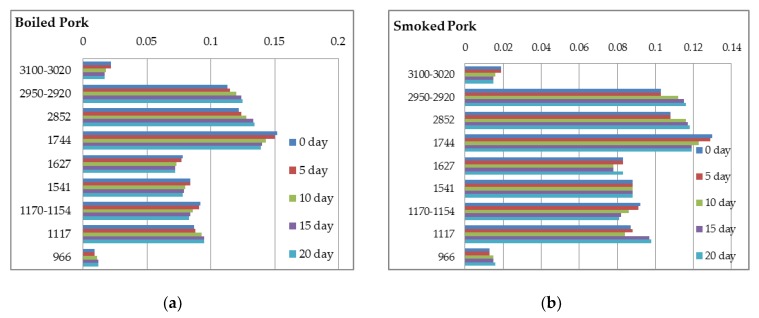

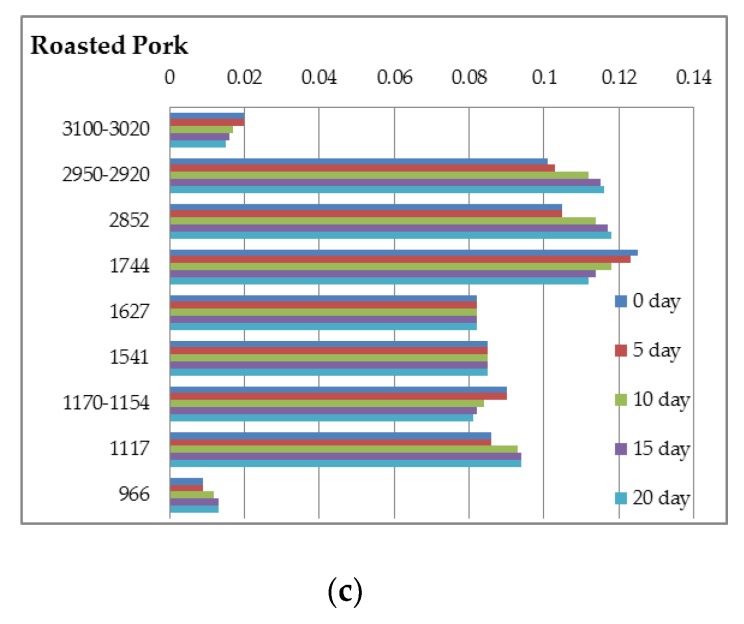
Spectral absorbance bands (intensities) (X-axis) at characteristic regions (cm^−1^) (Y-axis) during storage in refrigeration for twenty days: (**a**) boiled-pork hams; (**b**) smoked-pork hams; (**c**) roasted-pork hams.

In order to monitor the shelf-life of the studied ham samples towards chemical deterioration at a temperature of 4–6 °C, a comparative analysis was performed during storage in refrigeration for twenty days (Figure 5a,b and Figure 6a–c).

The most interesting findings from the FT-IR spectra interpretation are listed below. The intensities at 1744 and 1170–1154 cm^−1^, which are attributed to the C=O and the C-O stretching of carbonyl and ether bonds of triglycerides, respectively, were significantly (*p* < 0.05) decreased from day 10 until day 20 of refrigerated storage in all studied samples. Moreover, the intensities at 2950–2920, 2852, and 1117 cm^−1^, corresponding to the methyl and methylene groups’ stretching and deformation vibrations of fatty acids were significantly (*p* < 0.05) increased during the same storage period. These results probably indicate that refrigerated storage caused the degradation of triglycerides and the increase of free fatty acids as hydrolysis products. Therefore, refrigeration storage may inhibit the activity of triglycerol acyl hydrolases that catalyze lipid hydrolysis, releasing free fatty acids, and di- and monoglycerides [24].

The combined (*p* < 0.05) decrease in absorbance at 3100–3020 cm^−1^ with the increase in absorbance at 1117 cm^−1^ probably indicates the reduction of unsaturated fatty acids ratio over the saturated.

Another interesting finding was that the significant (*p* < 0.05) decrease of the intensities at 1627 and 1541 cm^−1^ was related to C=O stretching vibration and to the combined N-H bending vibration with the C-N stretching of proteins, respectively, from day 10 until day 20 of refrigerated storage in all studied samples. This result may be associated with a partial protein rupture.

Intensity at 966 cm^−1^, which is related to the out-of-plane deformation of C-H of trans-olefins, was significantly (*p* < 0.05) increased after 10 days of refrigerated storage in all studied samples. According to Marina et al. [25], the absorbance at 967 cm^−1^ increased as oxidation progressed, being an indicator of oxidative stability.

Refrigeration storage had a significant effect on color parameters of the final products (Table A1, Table A2, Table A3 and Table A4). It is apparent that, for all samples, the L* parameter was altered significantly throughout the preservation period. More specifically, the L* parameter of turkey hams presented an initial decrease from day 3 to day 6, stabilization until day 12, and an additional decrease from day 12 to day 15. Moreover, L* values of pork hams showed a similar trend, presenting a significant decrease at 15 days of storage. The hue (h) angle, which is related to a primary color (red, yellow, green, or blue) or the combination of two of them, varied with an opposite trend during the preservation period, increasing in turkey samples and decreasing in pork ones. Therefore, even though turkey hams revealed almost double h values compared to pork ones, with refrigeration storage, they tended to acquire more similar values. Yellowness (b*) increased significantly after 9–12 days of storage for all studied samples. Redness (a*) varied depending on meat type during storage. Therefore, a* values of turkey hams showed slight or significant increase, whereas a* values of pork hams significant decrease, throughout the preservation period. Color changes could be attributed to the dehydration of meat surface and to oxygen penetration into the tissues. Based on FT-IR spectra interpretation results, the color changes of hams could be the result of the oxidative degradation of lipids. According to Leygonie et al. [26] these color changes are undesirable and not reversible, deteriorating ham quality. An important conclusion resulting from the color measurement was that color parameters showed significant alterations after 9 days of refrigeration storage.

**Figure A1:**
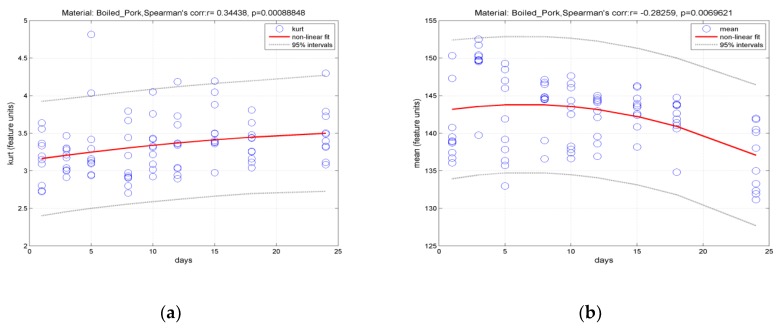
Variation of features (**a**) kurtosis and (**b**) mean intensity value of boiled pork hams during refrigerator storage.

Nonlinear regression analysis was employed on the computed features of boiled- and smoked-ham slices of pork and turkey in order to investigate variations in image texture with storage time. In boiled-pork ham (Figure A1), significant changes with time were observed in the kurtosis and mean-intensity features. Kurtosis values increased, while mean intensity values decreased with storage time. Kurtosis evaluates the shape of gray-level distribution. High kurtosis values indicate thin distributions around the mean with broad tails (leptokurtic), while low kurtosis values refer to distributions that are broad around the mean with light tails (platykurtic). Since kurtosis values increased with storage time, this indicates an increasing number of outlier pixels, i.e., large numbers of pixels with intensities spreading throughout the image gray-level spectrum. This redistribution of gray levels of pixels contributes to the loss of continuum of meaningful structures within the image. In boiled-pork ham, mean intensity value was found to decrease, indicating that boiled-pork-ham images turned darker with increasing storage time.

**Figure A2:**
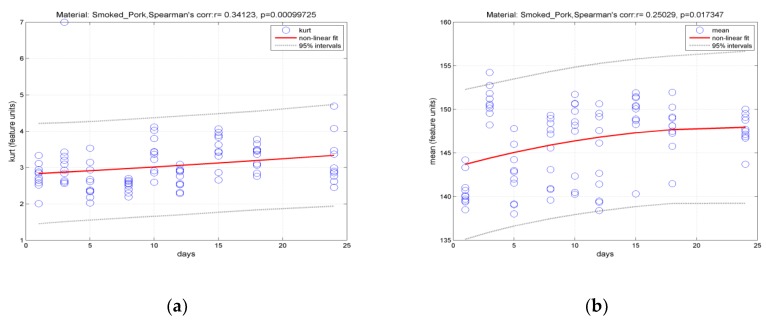
Variation of features (**a**) kurtosis and (**b**) mean intensity value of smoked pork hams during refrigerator storage.

In smoked-pork ham (Figure A2), there were four features that significantly varied with storage time: kurtosis, mean intensity, correlation, and gray-level nonuniformity. Kurtosis increased, signifying a decrease in textural continuum, and mean intensity increased, indicating smoked-pork-ham slices turned brighter. Correlation evaluates the level of linear dependencies among the gray levels of the image. The feature attained high values when there were meaningful structures present in the image. We found that correlation decreased with storage time in smoked-pork-ham slices. This indicates that structures in the smoked-pork slices broke down with storage time, losing textural continuum. Gray level nonuniformity was found to increase with storage time. The feature indicates that image structures are more unevenly distributed among the gray levels, probably due to redistribution of gray levels, resulting in the loss of structural continuum with storage time.

**Figure A3:**
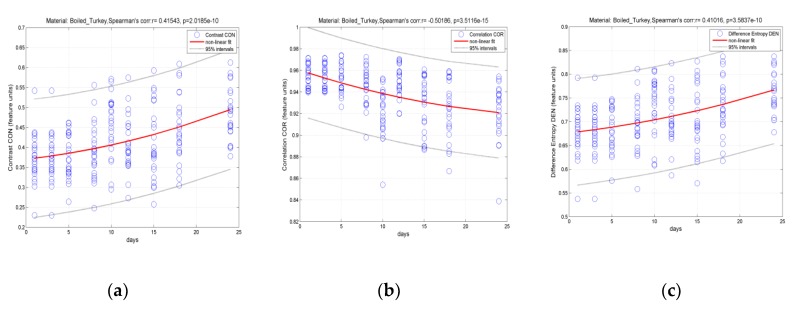

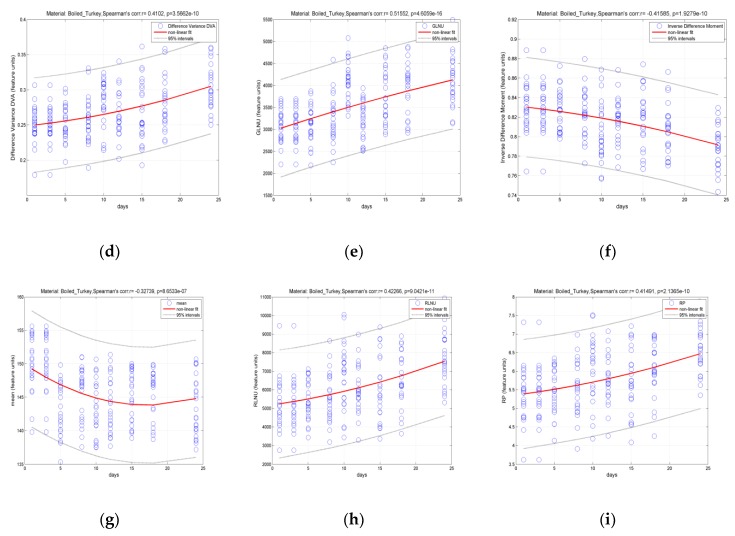
Variation of features (**a**) contrast, (**b**) correlation, (**c**) difference entropy, (**d**) difference variance, (**e**) great level non-uniformity, (**f**) inverse different moment, (**g**) mean intensity value, (**h**) run length non-uniformity and (**i**) run percentage of boiled turkey hams during refrigerator storage.

**Figure A4:**
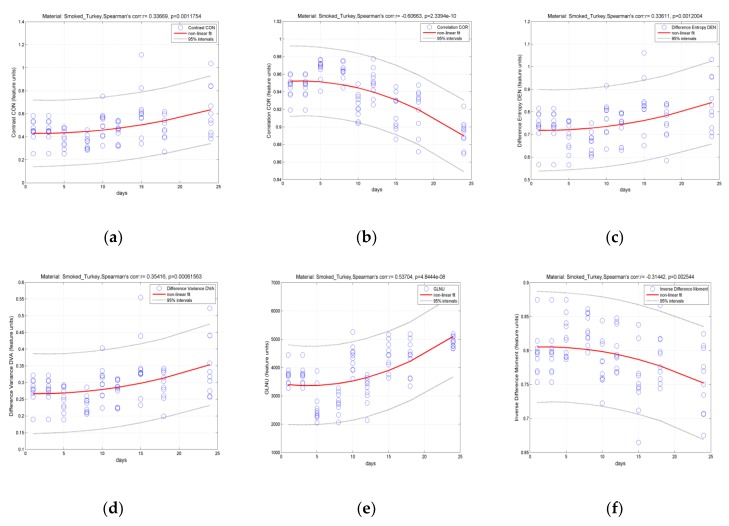

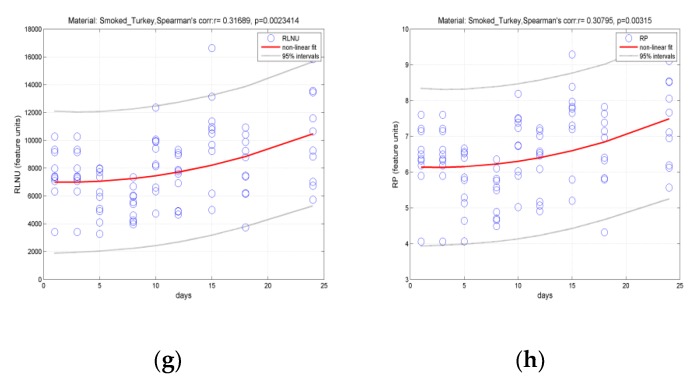
Variation of features (**a**) contrast, (**b**) correlation, (**c**) difference entropy, (**d**) difference variance, (**e**) great level non-uniformity, (**f**) inverse different moment, (**g**) run length non-uniformity and (**h**) run percentage of smoked turkey hams during refrigerator storage.

In boiled-turkey ham (Figure A3), with increasing storage time, image contrast increased, while image mean intensity and correlation decreased. This indicates that, with advancing storage time, boiled-turkey-ham images attained higher local contrast, their structural continuum deteriorated, and images became darker. Three more features from the co-occurrence matrix varied significantly with storage time: difference entropy, difference variance, and inverse-difference moment. Difference entropy evaluates variation of image lack of order. It was found to increase with storage time. Difference variance quantifies variation in image heterogeneity. It was found that the feature increased with storage time. Inverse-difference moment evaluates image homogeneity. It was found that it decreased with increasing storage time. The findings from the last three features indicate that, in boiled-turkey-ham slices, texture lost its homogeneity and structural order. Three more features, emanating from the run-length matrices, i.e., gray-level nonuniformity, run-length nonuniformity, and run percentage were found to increase significantly with storage time. Increase in gray-level nonuniformity indicates that structures were more unevenly distributed in the image gray levels. Increase in run-length nonuniformity indicates that image structures were more unevenly distributed in magnitude. Increase in run percentage indicates that nonlinearity in image structures increased. These findings indicate that, in boiled-turkey-ham slices, image structures lost continuum and local linearity, gray-level composition of image structures disintegrated, and sizes of structures changed. Similar findings were observed in smoked-turkey-ham slices (Figure A4) but for mean intensity, which seemed not to vary significantly with storage time.

Observing Figure A1 and Figure A2, it seems that textural-feature values of pork hams showed significant alterations after 9 days of refrigeration storage, while feature values of turkey hams presented continuous changes with storage time (Figure A3 and Figure A4).

## 4. Conclusions

In the present study, the combination of digital image analysis, color measurement, and FT-IR have been applied to classify hams according to meat type (pork and turkey), processing (boiled, smoked, roasted), and shelf life during refrigerated storage. Results showed that alterations of color parameters and textural features could be used to describe the differentiation among hams of different meat and processing type, and to evaluate ham quality during storage based on image changes of the surface of the ham slices. Moreover, FT-IR spectra interpretation revealed the profile of the main ingredients of the studied hams and their deterioration during storage.

## Figures and Tables

**Table 1:** Color parameters of turkey and pork hams.

	Turkey Boiled	Turkey Smoked	Pork Boiled	Pork Smoked	Pork Roasted
L*	66.76 ± 3.36 ^a^	62.99 ± 3.24 ^b^	54.41 ± 3.31 ^c^	56.51 ± 4.48 ^c^	58.20 ± 1.33 ^c^
C*	12.78 ± 1.80 ^ab^	11.92 ± 1.29 ^a^	13.02 ± 0.98 ^b^	14.67 ± 1.09 ^b^	17.42 ± 0.57 ^c^
h	60.27 ± 2.24 ^a^	66.08 ± 3.43 ^b^	36.66 ± 3.30 ^c^	34.76 ± 3.66 ^c^	35.85 ± 1.46 ^c^
ΔE*	45.59 ± 4.40 ^a^	41.63 ± 2.99 ^b^	34.88 ± 2.75 ^c^	37.76 ± 3.89 ^c^	40.03 ± 1.31 ^b^
a*	6.33 ± 0.48 ^a^	4.77 ± 0.82 ^b^	12.08 ± 0.89 ^c^	11.98 ± 0.85 ^c^	14.11 ± 0.44 ^d^
b*	11.12 ± 1.16 ^a^	10.91 ± 0.94 ^a^	7.74 ± 0.42 ^b^	8.33 ± 1.08 ^b^	10.19 ± 0.58 ^a^

Results represent means ± standard deviation (*n* = 30). Different letters after each value in the same row indicate statistically significant differences (*p* < 0.05).

**Table 2:** The spectral absorbance bands (intensities) of turkey- and pork-ham slices.

Regions	Turkey Boiled	Turkey Smoked	Pork Boiled	Pork Smoked	Pork Roasted
3500–3200	0.094 ± 0.002 ^a^	0.087 ± 0.003 ^b^	0.107 ± 0.002 ^c^	0.110 ± 0.004 ^c^	0.105 ± 0.003 ^c^
3100–3020	0.036 ± 0.002 ^a^	0.028 ± 0.003 ^b^	0.022 ± 0.002 ^c^	0.019 ± 0.002 ^c^	0.020 ± 0.002 ^c^
2950–2920	0.066 ± 0.002 ^a^	0.054 ± 0.001 ^b^	0.113 ± 0.004 ^c^	0.103 ± 0.002 ^d^	0.101 ± 0.002 ^d^
2852	0.080 ± 0.002 ^a^	0.068 ± 0.001 ^b^	0.122 ± 0.002 ^c^	0.108 ± 0.003 ^d^	0.105 ± 0.001 ^d^
1744	0.082 ± 0.003 ^a^	0.067 ± 0.003 ^b^	0.152 ± 0.004 ^c^	0.130 ± 0.003 ^d^	0.125 ± 0.002 ^d^
1627	0.064 ± 0.002 ^a^	0.051 ± 0.002 ^b^	0.078 ± 0.003 ^c^	0.083 ± 0.002 ^c^	0.082 ± 0.003 ^c^
1541	0.068 ± 0.003 ^a^	0.057 ± 0.001 ^b^	0.084 ± 0.004 ^c^	0.088 ± 0.002 ^c^	0.085 ± 0.005 ^c^
1450	0.077 ± 0.004 ^a^	0.064 ± 0.002 ^b^	0.098 ± 0.005 ^c^	0.099 ± 0.002 ^c^	0.097 ± 0.002 ^c^
1395	0.078 ± 0.001 ^a^	0.069 ± 0.003 ^b^	0.094 ± 0.003 ^c^	0.096 ± 0.003 ^c^	0.096 ± 0.002 ^c^
1240	0.056 ± 0.002 ^a^	0.051 ± 0.002 ^b^	0.096 ± 0.003 ^c^	0.092 ± 0.004 ^c^	0.091 ± 0.004 ^c^
1170–1154	0.067 ± 0.002 ^a^	0.060 ± 0.002 ^b^	0.092 ± 0.003 ^c^	0.092 ± 0.001 ^c^	0.090 ± 0.004 ^c^
1117	0.066 ± 0.003 ^a^	0.064 ± 0.001 ^a^	0.087 ± 0.002 ^b^	0.087 ± 0.004 ^b^	0.086 ± 0.002 ^b^
1040–1020	0.082 ± 0.001 ^a^	0.080 ± 0.001 ^a^	0.068 ± 0.001 ^b^	0.065 ± 0.003 ^b^	0.066 ± 0.003 ^b^
966	0.012 ± 0.001 ^a^	0.015 ± 0.001 ^b^	0.009 ± 0.001 ^c^	0.013 ± 0.001 ^ab^	0.009 ± 0.001 ^c^
870	0.032 ± 0.002 ^a^	0.031 ± 0.001 ^a^	0.048 ± 0.002 ^b^	0.049 ± 0.001 ^b^	0.049 ± 0.002 ^b^

Results represent means ± standard deviation (*n* = 30). Different letters after each value in the same row indicate statistically significant differences (*p* < 0.05).

## Appendix A

**Table A1:** Color parameters of boiled turkey hams during refrigerator storage.

Days	L*	h	a*	b*
0	66.76 ± 3.36 ^a^	60.27 ± 2.24 ^a^	6.33 ± 0.48 ^a^	11.18 ± 1.16 ^a^
3	64.22 ± 3.30 ^a^	60.62 ± 2.11 ^a^	6.32 ± 0,63 ^a^	10.92 ± 1.32 ^a^
6	61.99 ± 1.65 ^ab^	58.85 ± 1.45 ^ab^	6.21 ± 0,46 ^a^	11.06 ± 0.87 ^a^
9	58.37 ± 1.28 ^b^	57.09 ± 1.22 ^ab^	6.21 ± 0,38 ^a^	11.88 ± 0.61 ^ab^
12	55.95 ± 1.16 ^b^	56.75 ± 1.03 ^b^	6.13 ± 0.22 ^a^	12.75 ± 0.72 ^ab^
15	53.55 ± 1.07 ^c^	56.39 ± 0.68 ^b^	6.79 ± 0.31 ^a^	12.98 ± 0.60 ^b^
18	52.08 ± 0.73 ^c^	56.11 ± 1.34 ^b^	6.94 ± 0.29 ^a^	13.29 ± 0.57 ^b^
20	51.76 ± 0.81 ^c^	55.76 ± 1.06 ^b^	7.01 ± 0.36 ^a^	13.31 ± 0.49 ^b^

Results represent means ± standard deviation (*n* = 30). Different letters after each value in the same column indicate statistically significant differences (*p* < 0.05).

**Table A2:** Color parameters of smoked turkey hams during refrigerator storage.

Days	L*	h	a*	b*
0	62.99 ± 3.24 ^a^	66.08 ± 3.43 ^a^	4.77 ± 0.82 ^a^	10.91 ± 0.94 ^ab^
3	57.87 ± 1.56 ^a^	64.39 ± 2.12 ^a^	5.82 ± 0.67 ^ab^	10.65 ± 0.54 ^a^
6	54.75 ± 1.23 ^b^	64.34 ± 1.43 ^a^	5.72 ± 0.61 ^ab^	10.94 ± 0.68 ^ab^
9	54.49 ± 1.17 ^b^	62.75 ± 1.78 ^ab^	5.83 ± 0.39 ^ab^	11.86 ± 0.37 ^b^
12	54.34 ± 1.31 ^b^	62.73 ± 1.23 ^ab^	5.99 ± 0.44 ^ab^	12.75 ± 0.66 ^bc^
15	50.23 ± 1.06 ^c^	61.28 ± 0.56 ^b^	6.21 ± 0.26 ^b^	13.16 ± 0.58 ^c^
18	49.47 ± 0.82 ^c^	61.33 ± 0.45 ^b^	6.29 ± 0.29 ^b^	13.25 ± 0.41 ^c^
20	49.12 ± 1.28 ^c^	61.23 ± 0.71 ^b^	6.24 ± 0.32 ^b^	13.38 ± 0.52 ^c^

Results represent means ± standard deviation (*n* = 30). Different letters after each value in the same column indicate statistically significant differences (*p* < 0.05).

**Table A3:** Color parameters of boiled pork hams during refrigerator storage.

Days	L*	h	a*	b*
0	54.41 ± 3.31 ^a^	36.66 ± 3.30 ^a^	12.08 ± 0.89 ^a^	7.74 ± 0.42 ^a^
3	52.11 ± 1.34 ^a^	36.54 ± 2.79 ^a^	11.84 ± 0.59 ^a^	7.98 ± 0.51 ^a^
6	51.38 ± 1.13 ^a^	38.72 ± 1.75 ^a^	11.04 ± 0.28 ^a^	7.55 ± 0.61 ^a^
9	50.82 ± 1.08 ^a^	38.99 ± 1.58 ^a^	10.65 ± 0.75 ^ab^	7.92 ± 0.48 ^a^
12	48.81 ± 0.76 ^b^	39.11 ± 1.23 ^a^	10.65 ± 0.10 ^ab^	8.86 ± 0.33 ^b^
15	48.31 ± 0.85 ^b^	41.52 ± 1.02 ^b^	10.34 ± 0.28 ^b^	9.39 ± 0.71 ^b^
18	46.56 ± 0.68 ^c^	44.85 ± 1.31 ^c^	10.14 ± 0.19 ^b^	10.67 ± 0.64 ^c^
20	46.12 ± 0.89 ^c^	44.81 ± 0.69 ^c^	10.18 ± 0.22 ^b^	10.69 ± 0.46 ^c^

Results represent means ± standard deviation (*n* = 30). Different letters after each value in the same column indicate statistically significant differences (*p* < 0.05).

**Table A4:** Color parameters of smoked pork hams during refrigerator storage.

Days	L*	h	a*	b*
0	56.51 ± 4.48 ^a^	34.76 ± 3.66 ^a^	11.98 ± 0.85 ^a^	8.33 ± 1.08 ^a^
3	51.86 ± 2.47 ^ab^	34.57 ± 2.35 ^a^	11.03 ± 0.78 ^ab^	8.21 ± 0.78 ^a^
6	49.02 ± 1.54 ^b^	35.31 ± 2.01 ^a^	10.92 ± 0.66 ^ab^	8.85 ± 0.91 ^a^
9	48.64 ± 1.28 ^b^	39.31 ± 1.69 ^b^	10.82 ± 0.37 ^ab^	8.99 ± 0.76 ^a^
12	48.08 ± 1.72 ^b^	42.62 ± 1.32 ^c^	10.69 ± 0.65 ^ab^	9.38 ± 0.59 ^a^
15	47.97 ± 1.61 ^b^	44.27 ± 1.28 ^c^	10.11 ± 0.51 ^bc^	9.89 ± 0.80 ^ab^
18	42.94 ± 1.17 ^c^	47.34 ± 0.85 ^d^	9.47 ± 0.29 ^c^	10.85 ± 0.63 ^b^
20	42.97 ± 0.78 ^c^	47.31 ± 0.74 ^d^	9.49 ± 0.42 ^c^	10.95 ± 0.71 ^b^

Results represent means ± standard deviation (*n* = 30). Different letters after each value in the same column indicate statistically significant differences (*p* < 0.05).
